# The Effects of Food Security on Academic Performance of University Students at a Hispanic-Serving Institution

**DOI:** 10.3390/ijerph22020266

**Published:** 2025-02-12

**Authors:** Eva M. Moya, Gregory S. Schober, Amy Wagler, Jessica Ayala-Demeo Brown, Silvia M. Chavez-Baray, Panfeng Liang, Robbie Kennebrew

**Affiliations:** 1 Department of Social Work, The University of Texas at El Paso, El Paso, TX 79968, USA; 2 Rehabilitation Sciences Program, The University of Texas at El Paso, El Paso, TX 79968, USA; 3 Public Health Department, The University of Texas at El Paso, El Paso, TX 79968, USA; awagler2@utep.edu; 4 Department of Chicano Studies, Languages and Linguistics, The University of Texas at El Paso, El Paso, TX 79968, USA; 5 Border Biomedical Research Center, The University of Texas at El Paso, El Paso, TX 79968, USA; pliang@miners.utep.edu; 6 Interdisciplinary Health Sciences, The University of Texas at El Paso, El Paso, TX 79968, USA; rlkennebrew@miners.utep.edu

**Keywords:** food security, academic performance, Hispanic-Serving Institution, social determinants of health, barriers to academic success

## Abstract

Several studies find that low food security has negative effects on academic performance in higher education in the U.S., but the samples for these studies often have low percentages of Hispanic students. Consequently, it remains unclear if food security affects academic performance in predominantly Hispanic settings. Our study aims to analyze whether food security affects academic performance at a Hispanic-Serving Institution (HSI). Using original survey data collected on 2020 students enrolled at a large research-intensive HSI and cumulative logit models, we assess whether food security influences concentration and graduation delays among students at an HSI in the U.S.–Mexico border region. Our findings strongly suggest that low food security reduces concentration and increases delays for graduation at the HSI. The results have important implications for HSI leaders who want to improve student success, and we offer recommendations for future programs and investments to build student food security at HSIs. Because food security is a strong social determinant of health, the study is closely related to the topic of addressing social determinants of health to improve Hispanic health outcomes. When universities take action to build food security among HSI students, they simultaneously make an investment to improve Hispanic health outcomes.

## 1. Introduction

A large body of literature examines food security among students in higher education [1,2,3,4,5,6,7,8,9,10,11,12,13,14,15,16,17,18,19,20,21,22,23,24,25,26,27]. Food security is defined as “access by all people at all times to enough food for an active, healthy life” [28]. A lack of food security—or food insecurity—is a key challenge for many students at U.S. universities and colleges [1,3,9,20,21,22,25], and especially students at minority-serving institutions [6,19,29].

Within this literature, studies generally find that food insecurity reduces academic performance in higher education. Several studies find that food insecurity is associated with worse academic outcomes among higher education students, such as a lower grade point average (GPA), less frequent class attendance, reduced concentration in class and on assignments/exams, and delayed graduation or withdrawal [1,2,4,5,7,10,12,13,14,16,17,18,23,24,27,30,31].

The bulk of existing studies analyze the effects of food insecurity at institutions that have a low percentage of Hispanic students. The focus on predominantly non-Hispanic populations creates a gap in the literature, because the findings may not apply to Hispanic-Serving Institutions (HSIs) that serve an under-represented and disadvantaged social group in higher education. For example, there could be cultural factors that soften the impact of food insecurity on academic performance at HSIs, or HSIs may be taking additional actions to better identify and support food insecure students. Interestingly, Valle et al. [26] examined food insecurity and education outcomes at an HSI, and they found that food insecurity has no relationship with grades for Hispanic students. This surprising finding challenges whether the negative relationship between food insecurity and academic performance extends to predominantly Hispanic settings.

Our study aims to analyze the relationship between food insecurity and academic performance at another predominantly Hispanic university. By expanding the pool of evidence on this topic to another institutional setting, we aim to increase our understanding of student success barriers at Hispanic-Serving Institutions (HSIs) more broadly.

The study is closely related to the topic of addressing social determinants of health to improve Hispanic health outcomes. Food insecurity is an important social determinant of health that leads to a wide range of negative health effects [32,33,34,35]. Moreover, the completion of higher education is a main pathway to upward social mobility for low-income Hispanic students [36,37]. By understanding barriers to academic performance in higher education at HSIs, we also increase our understanding of ways to improve health outcomes among Hispanics.

## 2. Materials and Methods

### 2.1. Setting

This survey on food and housing security was conducted at an R1 university on the U.S.-Mexico border. The university has a majority Hispanic (Mexican American) student population primarily from the U.S.–Mexico border region, among the world’s most complex and dynamic regions. There are more than 25,000 students at the university, which practices an open enrollment policy. On campus, there is a food pantry that serves student food security needs, however many students do not know about or use the pantry services. The survey intends to broaden our knowledge about food and housing security, collect information about current status of food and housing security, and improve resources associated with food and housing security on campus.

### 2.2. Procedure

Online surveys were administered to students in 2022 via a university platform, Campus Labs, in order to collect, analyze, and translate data in real time. Current university students who were at least 18 years old were eligible to take the survey. University students had access to the survey in 1–21 November in Fall 2022, with a three-day survey extension to increase student engagement. During the study period, there were approximately 23,800 students meeting the eligibility criteria to participate in the study. The University Customer Relationship Management (CRM) sent four emails to eligible students, including an initial invitation and three weekly reminders. To participate in the survey, participants needed to complete a written informed consent form. No names or other personal identifiers were collected by researchers. Participants who voluntarily enrolled in the study consented electronically and completed the survey online. The anonymous survey contained 48 questions and took approximately 10 min to complete. Participants interested in being contacted for future studies were able to provide their preferred email address to the research team. This information was not linked to the initial survey. At the end of the survey, participants had the option to enter a drawing for five $50 gift cards for local grocery store. A total of 2020 participants—who met the inclusion criteria of being at least 18 years old and enrolled at the university at the time of study—completed the survey in 2022.

### 2.3. Analysis

Analysis is focused on examining the influence of students’ food security on their self-reported degree completion and academic concentration. Using the Six-Item Short Form Food Security Survey Module from the U.S. Department of Agriculture Economic Research Service [38], food security was categorized into three levels: very low, low, and marginal/high. The Six-Item Short Form Food Security Survey Module contains six survey questions related to household food security in the past year. For each respondent, the survey responses are coded based on the official scoring table and then added to create an index of household food security. The total index score for each respondent corresponds to one of the three food security levels described above.

To enhance the representativeness of our survey data with respect to the broader student population at the university, we employed a technique known as iterative proportional fitting, also referred to as raking in survey sampling. This process focused on four key factors: gender, ethnicity (whether respondents identified as Hispanic or not), enrollment type (part-time or full-time), and academic level (undergraduate, master’s, or doctoral). Before applying the raking procedure, we observed disparities between the characteristics of our survey participants and the demographics of the entire campus population. For instance, our survey respondents consisted of 85.40% full-time students and 14.60% part-time students, whereas the university’s overall enrollment included 63.50% full-time students and 36.50% part-time students.

During the raking process, the individual weights of survey respondents were adjusted to align the distribution of characteristics in our survey sample more closely with those of the overall population. This adjustment ensured that the marginal proportions of the survey data mirrored those of the broader university population. The raking was performed by the SAS macro developed by Izrael et al. [39].

Following the raking process, we recognized that certain individual-level factors exhibited an imbalance across different food security levels. Factors such as Federal Student Aid received in the past 12 months, household income, the number of dependents, and the current housing situation were identified as having a significant influence on an individual’s food security level. This imbalance could introduce bias into the modeling that is not reflective of the true associations in the data.

To address confounding variables that could be causing this imbalance and facilitate a more rigorous analysis, we employed Inverse Probability Weighting (IPW) with propensity scores. This approach is widely utilized in observational studies to establish causality when confounding or lurking variables are present. The concept of IPW for the analysis of three or more treatments was introduced by McCaffrey et al. [40]. For this study, propensity scores were computed through a multinomial logistic regression model applied to the raked data. In this model, food security level served as the response variable, while the confounding factors were treated as covariates. Subsequently, each survey respondent was assigned a weight equivalent to the inverse of their propensity score. This minimizes the impact of confounding factors so that the impact of food security level is less biased and robust. Additionally, by combining raking and IPW, we simultaneously correct for selection probabilities, nonresponse, and marginal discrepancies, producing a dataset that better reflects the target population. The approach also improves model performance, providing more accurate inferences.

Our assessments, both tabular and graphical, demonstrated a marked improvement in data balance after applying these weights. This adjustment helped mitigate the disparities in the distribution of individual-level factors across different food security levels, enhancing the validity of our analysis. This analysis is available upon request of the corresponding author.

After creating the PS-weighted data, cumulative logit models with proportional odds for three ordinal response variables were estimated on the three response variables outlined in Table 1.

The cumulative logit models with proportional odds summarize the relationships between these response variables and the exposure (food security) and control variates (gender, ethnicity, income, housing security, and financial aid), while accounting for the ordinal nature of the responses. This flexible and informative approach allows us to make meaningful inferences about how food security and other factors are associated with academic outcomes.

## 3. Results

### 3.1. Descriptive Statistics

Table 2 provides summary data on the levels of food security among the population-adjusted data used for the study. Note that only 39% of students reported marginal or high levels of food security; hence, 61% were experiencing difficulties with food security.

Table A1 in Appendix A provides a breakdown of the demographic information by food security level as presented in Table 1. Note that all factors, excluding gender, being white non-Hispanic, and enrollment level, included in the study are statistically significant using univariate tests of association. This provides additional evidence of the utility of using a multivariable approach for analysis since many of these factors are likely to confound the associations between food security and the academic outcomes.

**Figure 1:**
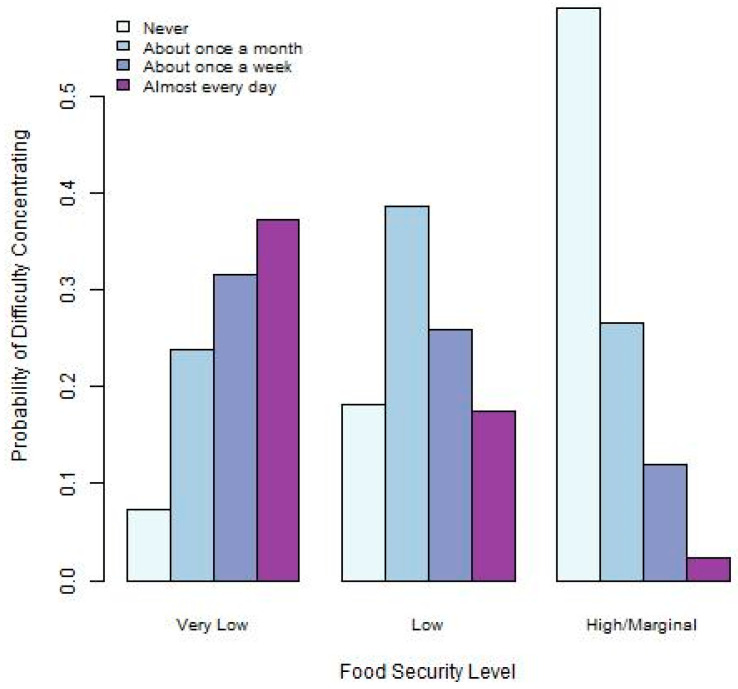
Barplot depicting relationship between food security and difficulty concentrating.

**Figure 2:**
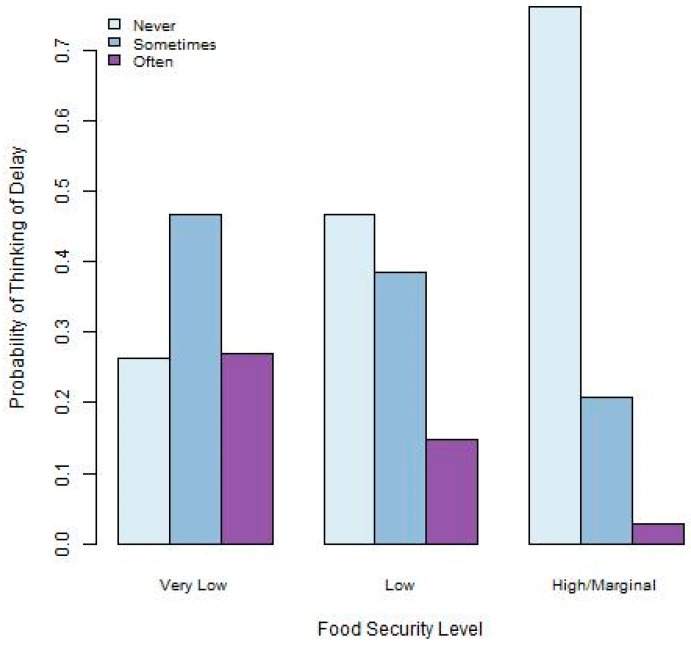
Barplot depicting relationship between food security and thinking of delay.

**Figure 3:**
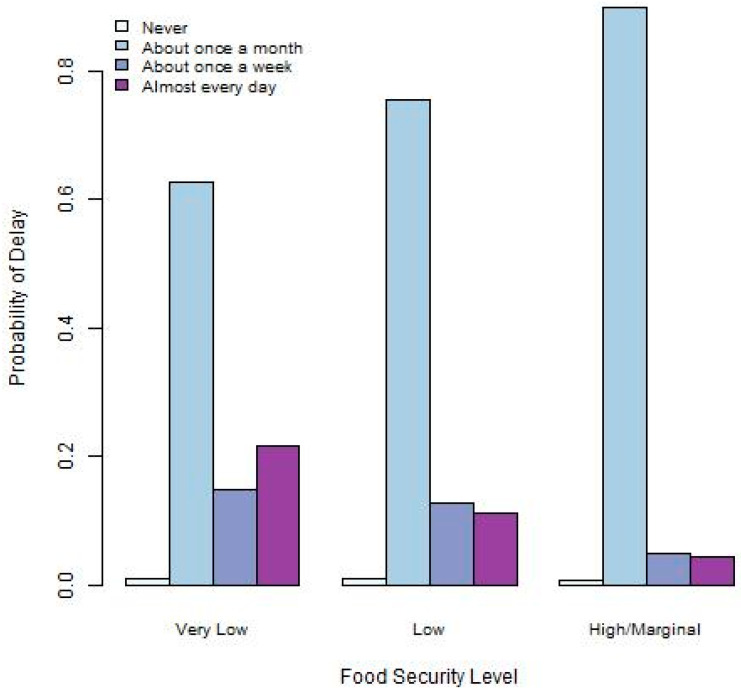
Barplot depicting relationship between food security and degree delay.

Graphical visualizations of the relationships between food security and the three academic outcomes provide some insight into how these may be associated. Figure 1, Figure 2 and Figure 3 provide summaries for the relationships between food security and difficulty concentrating, thinking of a degree delay, and taking a degree delay. Figure 1 indicates that those at the lowest levels of food security experience the highest levels of difficulty concentrating on academic pursuits. Figure 2, depicting the relationship between food security and thinking of a delay, indicates that those with the lowest food security also have the highest frequency of thinking of degree delays. Finally, Figure 3 indicates that the highest levels of delaying their degree take place among those with lower levels of food security.

### 3.2. Model Results

All model results are provided in detail in Appendix A Table A2, Table A3 and Table A4 for the primary study outcomes of difficulty concentrating, thinking of delay, and degree delay, respectively. All models for each endpoint used the same set of explanatory variables to separately model the associations between the outcome and explanatory variables. An analysis of the results appears in this section, along with forest plots depicting model results.

#### 3.2.1. Difficulty Concentrating

Low food security was associated with poor concentration among students. The odds of experiencing concentration difficulties were more than four times as high in the “low” food security group compared to that in the “marginal/high” food security group (OR = 4.342, 95% CI [3.219, 5.858]), and more than eleven times as high in the “very low” food security group compared to that in the “high” food security group (OR = 11.149, 95% CI [8.139, 15.274]). The control variables that impacted concentration in the model include the following:

- Black students were more likely to experience concentration challenges than non-black students (OR = 1.934, 95% CI [1.032, 3.627]);
- A higher income (above $50,000) was associated with a reduced likelihood of concentration problems (OR = 0.530, 95% CI [0.388, 0.725]);
- Students who did not have a stable residential space and often had to spend the night elsewhere seemed to have poorer concentration than students who had a permanent address (OR = 3.491, 95% CI [1.429, 8.527]);
- Students who received student aid or emergency food within the last 12 months had concentration problems more often than those who did not receive these (OR = 1.380, 95% CI [1.008, 1.889] and OR = 1.696, 95% CI [1.306, 2.202]).

**Figure 4:**
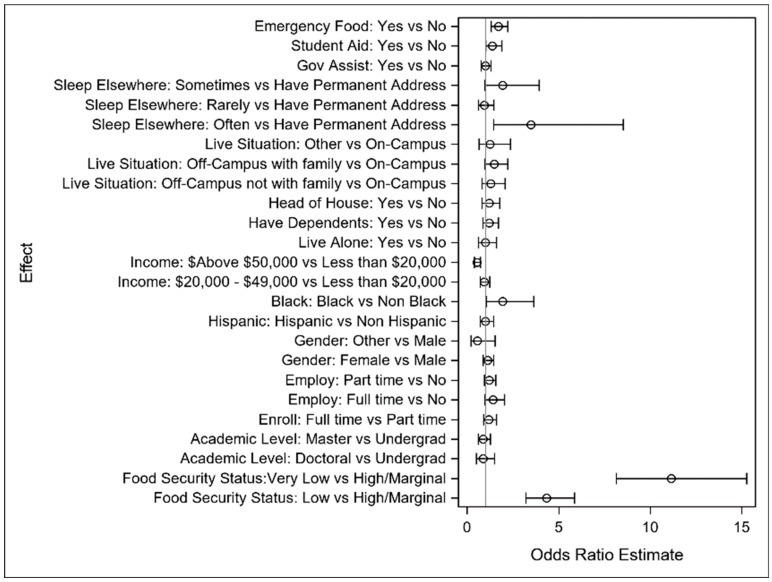
Forest plot of odds ratio and interval estimates for difficulty concentrating.

Figure 4 visually depicts the logistic model results. Notice that the impact of food security, both very low and low levels versus high, have a much larger impact on the outcome than the control variables, emphasizing the magnitude of impact of food security problems relative to other common risk factors in this context.

#### 3.2.2. Thinking of Delay

Both “low” and “very low” food security increased the likelihood of students thinking about extending their academic programs. In the “very low” food security group, the odds of “often thought about delay” were more than six times higher than that in the “marginal/high” food security group (OR = 6.560, 95% CI [4.779, 9.006]), and, in the “low” food security group, the odds were around twice as high (OR = 3.041, 95% CI [2.210, 4.185]). The other factors impacting thinking about a degree delay include the following:

- Students with full-time jobs were more likely to consider delaying their degree completion compared to students without employment (OR = 2.003, 95% CI [1.363, 2.944]);
- Students living on campus were more likely to stay on track with their degree completion;
- Students who received emergency food within the last 12 months thought about a delay more often than those who did not receive it (OR = 2.022, 95% CI [1.522, 2.685]).

**Figure 5:**
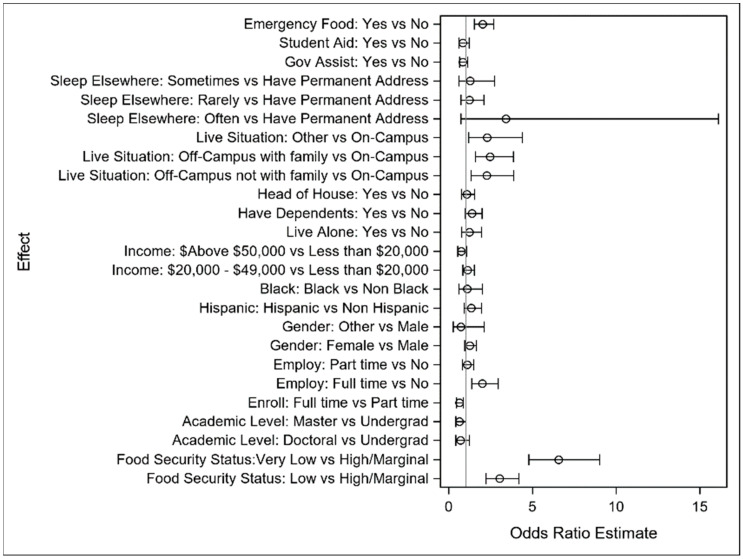
Forest plot of odds ratio and interval estimates for thinking of delay.

Figure 5, depicting the logistic regression model results, indicates the relative impact of food security on thinking of a delay. As before, there is an outsized impact of a lack of food security on thinking of delaying academic programs relative to other well-established risk factors.

#### 3.2.3. Degree Delay

Students with “very low” food security were significantly more likely to delay their degree completion compared to those with “marginal/high” food security (OR = 4.820, 95% CI [3.175, 7.319]). The odds of a delay for the “low” food security group were doubled compared to those for the “marginal/high” food security groups (OR = 1.954, 95% CI [1.238, 3.084]). Other factors impacting a degree delay include the following:

- Doctoral and master’s students were less likely to delay degree completion compared to undergraduate students (OR = 0.332, 95% CI [0.162, 0.680] and OR = 0.362, 95% CI [0.199, 0.659]);
- Full-time enrollment decreased the odds of extending academic programs (OR = 0.499, 95% CI [0.346, 0.719]);
- Being full-time-employed increased the odds of extending academic programs (OR = 1.667, 95% CI [1.056, 2.631]);
- Having dependents increased the odds of delaying degree completion (OR = 1.954, 95% CI [1.268, 3.010]);
- Living on campus decreased the odds of delaying degree completion;
- Students who received emergency food within the last 12 months were more likely to delay than those who did not receive it (OR = 1.500, 95% CI [1.058, 2.127]).

**Figure 6:**
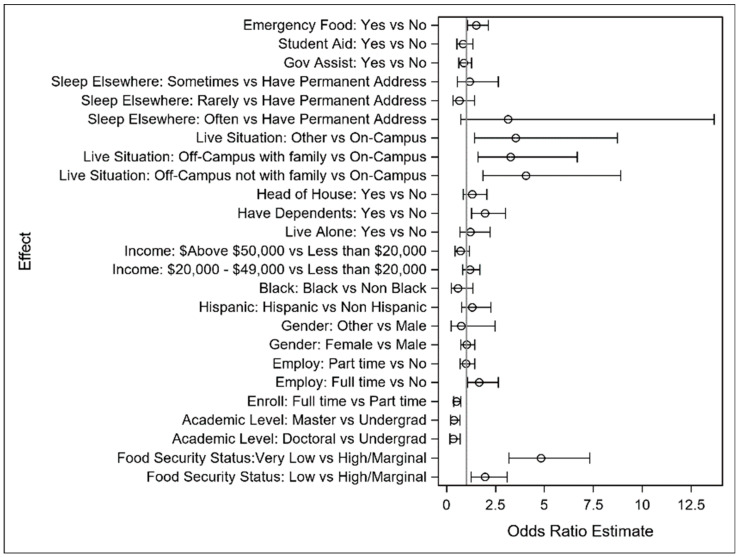
Forest plot of odds ratio and interval estimates for degree delay.

In Figure 6, the logistic regression model results are visually depicted. As with the other academic outcomes, there is a significantly larger impact of a lack of food security on degree completion versus other risk factors, especially for those with very low levels of food security.

This study has two main limitations. First, due to the nature of the data collection as a volunteer response and the likelihood of students experiencing food security issues having a higher propensity to respond, we acknowledge that there may be some bias towards the students with food security problems. However, we took every effort to correct for this bias in the data by weighting results to reflect overall student demographics, including potential confounding factors in the multivariate models, and employing inverse probability weighting to adjust for the confounding variable impacts on the observed relationships between food security and the three academic outcomes. Second, the relatively large percentage of Hispanic students in the sample (82.5%) limits our ability to evaluate the role that ethnicity plays in moderating the effects of food security on academic performance.

## 4. Discussion

This study aimed to analyze the relationship between food security and academic outcomes among college students and, specifically, was designed to increase the understanding of student success barriers at an HSI. Academic outcomes in this case were defined as difficulty concentrating due to concerns about food or other necessities, thinking about delaying the completion of their degree due to a lack of money for essentials, and delaying their degree because of financial constraints related to food, rent, or other necessities. Our study approaches the topic of food security as a social determinant of health (non-medical driver of health) and suggests that the effects of food insecurity led to poor academic outcomes, which then limits the upward social mobility of Hispanic students. The findings of the study suggest that low/very low food security was associated with poor concentration among students; low/very low food security increased the likelihood of students thinking about extending their academic program; and students experiencing low/very low food security were more likely to delay their degree completion.

Consistent with the existing literature [7], this study revealed key understandings in the relationship between food insecurity and housing stability, income or employment status, and race/ethnicity, as well as academic outcomes. A student’s level of food security is heavily influenced by other factors, making them either more or less susceptible to experiencing hardships, especially in academic settings. Students in this study who reported receiving financial aid or emergency food within the last 12 months were more likely to experience difficulties with concentration in the academic setting due to their concerns about food, rent, or other necessities. These students also were more likely to delay the completion of their academic program. Although Valle et al. [26] found that food insecurity had no relationship with grades for Hispanic students, the role that income played in food security was acknowledged, in that students with a lower income had a higher likelihood of having poor grades. Our study complements this observation, where students with a higher income were less likely to experience concentration problems. Future work should examine the relationship between Hispanic students and academic performance to reinforce the established findings.

A noticeable trend regarding a student’s housing situation emerged in our findings. Specifically, students living on-campus were more likely to stay on track to complete their degree and the likelihood of delaying their degree plan was decreased. This observation supports earlier findings [7] that identified living on-campus as a protective factor for food security, as off-campus living can impact a students’ finances more due to the need for reliable transportation and accessing affordable food. A recent survey of Latinos in Higher Education [41] found that 85 percent of Latino college students reported a lack of access to affordable and healthy food. In addition, two-thirds of Latino students considered leaving school at some point during their time in college, with one-third taking a leave of absence at some point. Subsequent work needs to focus on the relationship between on- and off-campus housing and the accessibility to services and food to lend additional validity to the existing claims.

This study explores a comprehensive list of adverse effects that are associated with low food security. However, this information only represents the impacts at one HSI; therefore, additional studies across other HSIs may be needed to confirm the impact on academic outcomes for Hispanic students.

In the U.S.–Mexico border region, our findings suggest that university students experience difficulties with food security, which impact their learning and time to graduation. These negative academic outcomes carry an increased economic and social cost at the local, state, and national level. These findings add to the burdens of the non-medical drivers of health, thus increasing the risk of health conditions and decreasing life expectancy. It is important to understand and address the challenges that the students face. Students are delaying their degree because they need to work to afford food, which in turn has costs for the university, city, and community. It is important to rethink the ways we can bring nutritious, accessible, and affordable food to university students.

Achieving high food security for all students will reduce the costs of education and the risks of chronic conditions, thus increasing the productivity and well-being of the population. Elevating food security in the university may result in a win–win for everyone.

To build food security for all students, universities need to understand the challenges that students face; collect data on the scope of the challenges and key barriers to success; work together with students to design and implement action programs; increase financial support for food security efforts; elevate education in food and housing security; and partner with government organizations, companies, and non-profit organizations to increase the stability of the population and have healthy and productive people.

### 4.1. Broader Implications

The findings from this study have broader implications for the long-term socioeconomic mobility of Hispanic residents in the U.S. Higher education represents a key pathway to upward social mobility for low-income Hispanic individuals and families [36,37]. Because food insecurity creates barriers for academic performance and educational attainment, food-insecure Hispanic students at HSIs face increased challenges to secure upward social mobility via higher education. Investments to address student food insecurity at HSIs, therefore, serve as investments to advance long-term socioeconomic mobility among Hispanics.

When working together with students to address barriers to student success at HSIs, HSI administrators should consider making investments in food assistance. Students possess important expertise on the challenges related to student food security, and administrators need to listen and follow the guidance of students when deciding which programs to develop or expand. For example, in line with previous research [15], our survey revealed that few students use the food pantry, and stigma is a key barrier for food pantry use. When designing food assistance programs at HSIs, efforts need to be made to ensure that food assistance is shared in a dignifying manner that minimizes stigma. Moreover, other types of assistance resources need to be expanded at HSIs, because food insecurity often overlaps with other forms of basic needs insecurity [19]. A wraparound service approach, which offers multiple types of assistance in a coordinated manner and can be applied at schools [42], is a promising model to help address the basic needs insecurity of students. Food literacy and financial literacy training should also be offered at HSIs, so that students can maximize any resources that they have [15].

At HSIs, some students may not be eligible for government food assistance programs—such as the Supplemental Nutrition Assistance Program (SNAP)—due to immigration status or other factors. HSI administrators should, therefore, invest in a comprehensive set of assistance resources that are available to all students, rather than only investing in more workshops on how to enroll in government programs.

The recommendations described above are relevant to institutions beyond HSIs. To advance student success in higher education settings with high rates of food insecurity, it is important for administrators to seek student-led solutions, expand food assistance offerings, and consider the wraparound service approach to address related forms of basic needs insecurity. The recommendation to offer comprehensive assistance options for all students—regardless of immigration status—is also applicable to institutions beyond HSIs. Even if an institution does not technically qualify as an HSI, they still may have a sizable number of students who are not eligible for government food assistance programs.

### 4.2. Limitations

The study has three main limitations. First, the cross-sectional nature of the survey data limits our ability to make causal inferences. Second, the relatively large percentage of Hispanic students in the sample (82.5%) limits our ability to evaluate the role that ethnicity plays in moderating the effects of food security on academic performance. Third, the presence of missing data may introduce a non-response bias in the results, since those who did not respond to some items may differ significantly to the general population.

## 5. Conclusions

This study highlights the significant barriers that food insecurity poses to academic success for university students at HSIs. The findings reveal a strong association between low food security and negative academic outcomes, including poor concentration and delayed degree completion. These challenges, often compounded by financial and housing instability, hinder students’ academic progress and limits their access to socioeconomic advancement. Addressing food insecurity can reduce educational costs, improve health outcomes, and enhance overall student well-being and productivity.

For universities to effectively support students, it is crucial that we understand the unique challenges faced by HSI populations. Collaborative efforts between universities, researchers, and policymakers are essential in identifying the underlying determinants of food insecurity and developing targeted solutions. Universities should prioritize data-driven approaches to create comprehensive support systems that address not only food security but also housing and financial stability. Building partnerships with local communities, government agencies, and non-profit organizations can further strengthen these efforts and promote long-term stability for students. Universities can take several concrete actions to build food security at HSIs, including efforts to expand food assistance resources for all students, develop wraparound services, and increase awareness of available resources and eligibility requirements (when applicable).

Future research should continue to explore the intersectionality of food security, housing, income, and academic outcomes to develop more effective strategies for supporting students. More research is needed on basic needs insecurity at HSIs and other minority-serving institutions. Longitudinal studies especially are needed to help gain empirical leverage to make estimates of causal effects over time. Universities must also take proactive steps to address these key social determinants of health, cultivating an environment that promotes both academic achievement and student well-being. By addressing food insecurity and its related challenges, higher education institutions can contribute to the development of healthier, more resilient student populations, with positive implications for society at large. In the long term, investments in student food security will produce more effective leaders in society who are better equipped to solve pressing community needs.

## Figures and Tables

**Table 1:** Response variables for logistic regression models on academic outcomes.

Response	Description	Levels
**Difficulty Concentrating**	This variable assesses the frequency with which students experience poor concentration at school due to concerns about food, rent, or other necessities.	Never About once a month About once a week Almost every day
**Thinking of Delay**	This variable evaluates how often students think about delaying the completion of their degree due to a lack of money for essentials.	Never Sometimes Often
**Delayed Degree**	This variable shows whether students have delayed their degree because of financial constraints related to food, rent, or other necessities.	No delay Delay by one semester Delay by two semesters

**Table 2:** Food security levels frequencies and relative frequencies.

	Frequency	Relative Frequency
**Very Low**	604	34.93
**Low**	452	26.14
**High/Marginal**	673	38.92

## Data Availability

The datasets generated and analyzed during the current study are available from the corresponding author upon reasonable request.

## Appendix A

**Table A1:** Demographic variables by food security status.

	Food Security Status				
	Very Low (*N* = 604)	Low (*N* = 452)	High/Marginal (*N* = 673)	Total (*N* = 1729)	*p*-Value
Physical PROMIS Scale					<0.0001 ^1^
*n*	597	447	665	1709	
Mean (SD)	6.8 (1.63)	7.2 (1.63)	7.8 (1.53)	7.3 (1.64)	
Median	7.0	7.0	8.0	8.0	
Range	2.0, 10.0	2.0, 10.0	2.0, 10.0	2.0, 10.0	
Mental PROMIS Scale					<0.0001 ^1^
*n*	597	447	665	1709	
Mean (SD)	5.3 (1.90)	5.8 (1.89)	6.3 (1.94)	5.8 (1.96)	
Median	5.0	6.0	6.0	6.0	
Range	2.0, 10.0	2.0, 10.0	2.0, 10.0	2.0, 10.0	
UTEP Enrollment, n (%)					0.6775 ^2^
Full time	519 (85.9%)	380 (84.1%)	576 (85.6%)	1475 (85.3%)	
Part time	85 (14.1%)	72 (15.9%)	97 (14.4%)	254 (14.7%)	
Employed, n (%)					0.4446 ^2^
Full time	124 (20.5%)	79 (17.5%)	132 (19.6%)	335 (19.4%)	
Part time	287 (47.5%)	216 (47.8%)	299 (44.4%)	802 (46.4%)	
No	193 (32.0%)	157 (34.7%)	242 (36.0%)	592 (34.2%)	
Age Group, n (%)					<0.0001 ^2^
Under 18	1 (0.5%)	0 (0.0%)	1 (0.4%)	2 (0.4%)	
18–24	120 (65.2%)	96 (64.4%)	180 (80.7%)	396 (71.2%)	
25–34	29 (15.8%)	40 (26.8%)	26 (11.7%)	95 (17.1%)	
35–44	16 (8.7%)	3 (2.0%)	9 (4.0%)	28 (5.0%)	
45–64	18 (9.8%)	9 (6.0%)	7 (3.1%)	34 (6.1%)	
65 and above	0 (0.0%)	1 (0.7%)	0 (0.0%)	1 (0.2%)	
Missing	420	303	450	1173	
Hispanic, n (%)					0.1714 ^2^
Non-Hispanic	119 (19.7%)	77 (17.0%)	106 (15.8%)	302 (17.5%)	
Hispanic	485 (80.3%)	375 (83.0%)	567 (84.2%)	1427 (82.5%)	
Black, n (%)					0.0009 ^2^
Non-Black	563 (93.2%)	434 (96.0%)	656 (97.5%)	1653 (95.6%)	
Black	41 (6.8%)	18 (4.0%)	17 (2.5%)	76 (4.4%)	
White, n (%)					0.4245 ^2^
Non-White	514 (85.1%)	381 (84.3%)	555 (82.5%)	1450 (83.9%)	
White	90 (14.9%)	71 (15.7%)	118 (17.5%)	279 (16.1%)	
Gender, n (%)					0.2811 ^2^
Female	400 (66.2%)	309 (68.4%)	432 (64.2%)	1141 (66.0%)	
Male	187 (31.0%)	132 (29.2%)	230 (34.2%)	549 (31.8%)	
Other	17 (2.8%)	11 (2.4%)	11 (1.6%)	39 (2.3%)	
Income in 2022, n (%)					<0.0001 ^2^
Less than $20,000	281 (46.5%)	180 (39.8%)	181 (26.9%)	642 (37.1%)	
$20,000–$49,000	232 (38.4%)	182 (40.3%)	237 (35.2%)	651 (37.7%)	
$Above $50,000	91 (15.1%)	90 (19.9%)	255 (37.9%)	436 (25.2%)	
Academic Level, n (%)					0.8097 ^2^
Undergrad	465 (77.0%)	361 (79.9%)	531 (78.9%)	1357 (78.5%)	
Master’s	86 (14.2%)	59 (13.1%)	90 (13.4%)	235 (13.6%)	
Doctoral	53 (8.8%)	32 (7.1%)	52 (7.7%)	137 (7.9%)	
Live Alone, n (%)					0.0021 ^2^
Yes	85 (14.1%)	59 (13.1%)	55 (8.2%)	199 (11.5%)	
No	519 (85.9%)	393 (86.9%)	618 (91.8%)	1530 (88.5%)	
Number of Dependents, n (%)					<0.0001 ^2^
1	52 (66.7%)	53 (94.6%)	54 (91.5%)	159 (82.4%)	
4 or more	26 (33.3%)	3 (5.4%)	5 (8.5%)	34 (17.6%)	
Missing	526	396	614	1536	
Head of Household, n (%)					<0.0001 ^2^
Yes	206 (34.1%)	117 (25.9%)	125 (18.6%)	448 (25.9%)	
No	398 (65.9%)	335 (74.1%)	548 (81.4%)	1281 (74.1%)	
Living Situation, n (%)					<0.0001 ^2^
On-Campus	73 (12.1%)	41 (9.1%)	31 (4.6%)	145 (8.4%)	
Off-Campus with family	370 (61.3%)	323 (71.5%)	527 (78.3%)	1220 (70.6%)	
Off-Campus not with family	116 (19.2%)	62 (13.7%)	79 (11.7%)	257 (14.9%)	
Other	45 (7.5%)	26 (5.8%)	36 (5.3%)	107 (6.2%)	
Sleeping Elsewhere, n (%)					<0.0001 ^2^
Often	12 (2.0%)	5 (1.1%)	2 (0.3%)	19 (1.1%)	
Sometimes	27 (4.5%)	9 (2.0%)	4 (0.6%)	40 (2.3%)	
Rarely	48 (8.0%)	28 (6.2%)	34 (5.1%)	110 (6.4%)	
Have Permanent Address	516 (85.6%)	410 (90.7%)	633 (94.1%)	1559 (90.2%)	
Missing	1	0	0	1	
Government Assistance, n (%)					<0.0001 ^2^
Yes	255 (42.2%)	178 (39.4%)	206 (30.6%)	639 (37.0%)	
No	349 (57.8%)	274 (60.6%)	467 (69.4%)	1090 (63.0%)	
Federal Student Aid, n (%)					0.0007 ^2^
Yes	491 (81.3%)	379 (83.8%)	505 (75.0%)	1375 (79.5%)	
No	113 (18.7%)	73 (16.2%)	168 (25.0%)	354 (20.5%)	
Emergency Food, n (%)					<0.0001 ^2^
Yes	284 (47.0%)	168 (37.2%)	101 (15.0%)	553 (32.0%)	
No	320 (53.0%)	284 (62.8%)	572 (85.0%)	1176 (68.0%)	

^1^ Kruskal–Wallis *p*-value; ^2^ Chi-Square *p*-value.

**Table A2:** Model results for difficulty concentrating outcome.

Factor	Odds Ratio	95% Confidence Limits	
Food Security: Low vs. High/Marginal	4.342	3.219	5.858
Food Security: Very Low vs. High/Marginal	11.149	8.139	15.274
Doctoral vs. Undergrad	0.859	0.492	1.501
Master’s vs. Undergrad	0.867	0.594	1.265
Work: Full time vs. Part time	1.187	0.883	1.595
Enrollment: Full time vs. No	1.410	0.975	2.039
Enrollment: Part time vs. No	1.198	0.922	1.556
Female vs. Male	1.119	0.867	1.445
Other vs. Male	0.551	0.201	1.512
Hispanic vs. Non-Hispanic	1.001	0.700	1.432
Black vs. Non-Black	1.934	1.032	3.627
Income: $20,000–$49,000 vs. <$20,000	0.936	0.711	1.233
Income: >$50,000 vs. <$20,000	0.530	0.388	0.725
Lives Alone	1.001	0.626	1.601
Has Dependents	1.196	0.842	1.698
Head of Household	1.201	0.816	1.768
Living Situation: Off-Campus not with family vs. On-Campus	1.282	0.799	2.057
Living Situation: Off-Campus with family vs. On-Campus	1.470	0.983	2.198
Living: Situation: Other vs. On-Campus	1.237	0.649	2.356
Housing: Often vs. Have Permanent Address	3.491	1.429	8.527
Housing: Rarely vs. Have Permanent Address	0.941	0.609	1.455
Housing: Sometimes vs. Have Permanent Address	1.931	0.953	3.916
Government Assistance	0.989	0.762	1.283
Student Aid	1.380	1.008	1.889
Emergency Food	1.696	1.306	2.202

NOTE: The degrees of freedom in computing the confidence limits are 1713.

**Table A3:** Model results for thinking of delay outcome.

Factor	Odds Ratio	95% Confidence Limits	
Food Security: Low vs. High/Marginal	3.041	2.210	4.185
Food Security: Very Low vs. High/Marginal	6.560	4.779	9.006
Doctoral vs. Undergrad	0.697	0.400	1.214
Master’s vs. Undergrad	0.636	0.404	1.001
Work: Full time vs. Part time	0.623	0.452	0.860
Enrollment: Full time vs. No	2.003	1.363	2.944
Enrollment: Part time vs. No	1.089	0.810	1.464
Female vs. Male	1.242	0.943	1.637
Other vs. Male	0.722	0.247	2.113
Hispanic vs. Non-Hispanic	1.334	0.907	1.962
Black vs. Non-Black	1.085	0.588	2.002
Income: $20,000–$49,000 vs. <$20,000	1.124	0.838	1.508
Income: >$50,000 vs. <$20,000	0.737	0.514	1.057
Lives Alone	1.225	0.767	1.957
Has Dependents	1.389	0.983	1.964
Head of Household	1.073	0.747	1.541
Living Situation: Off-Campus not with family vs. On-Campus	2.262	1.319	3.877
Living Situation: Off-Campus with family vs. On-Campus	2.467	1.580	3.850
Living: Situation: Other vs. On-Campus	2.273	1.180	4.378
Housing: Often vs. Have Permanent Address	3.416	0.725	16.094
Housing: Rarely vs. Have Permanent Address	1.226	0.723	2.081
Housing: Sometimes vs. Have Permanent Address	1.262	0.584	2.729
Government Assistance	0.830	0.624	1.105
Student Aid	0.838	0.587	1.194
Emergency Food	2.022	1.522	2.685

NOTE: The degrees of freedom in computing the confidence limits are 1713.

**Table A4:** Model results for degree delay outcome.

Factor	Odds Ratio	95% Confidence Limits	
Food Security: Low vs. High/Marginal	1.954	1.238	3.084
Food Security: Very Low vs. High/Marginal	4.820	3.175	7.319
Doctoral vs. Undergrad	0.332	0.162	0.680
Master’s vs. Undergrad	0.362	0.199	0.659
Work: Full time vs. Part time	0.499	0.346	0.719
Enrollment: Full time vs. No	1.667	1.056	2.631
Enrollment: Part time vs. No	0.989	0.679	1.439
Female vs. Male	1.021	0.727	1.435
Other vs. Male	0.736	0.220	2.462
Hispanic vs. Non-Hispanic	1.309	0.758	2.259
Black vs. Non-Black	0.572	0.244	1.340
Income: $20,000–$49,000 vs. <$20,000	1.184	0.824	1.703
Income: >$50,000 vs. <$20,000	0.697	0.425	1.146
Lives Alone	1.208	0.659	2.212
Has Dependents	1.954	1.268	3.010
Head of Household	1.307	0.830	2.058
Living Situation: Off-Campus not with family vs. On-Campus	4.051	1.843	8.905
Living Situation: Off-Campus with family vs. On-Campus	3.268	1.599	6.681
Living: Situation: Other vs. On-Campus	3.523	1.422	8.728
Housing: Often vs. Have Permanent Address	3.136	0.719	13.678
Housing: Rarely vs. Have Permanent Address	0.655	0.304	1.411
Housing: Sometimes vs. Have Permanent Address	1.177	0.526	2.632
Government Assistance	0.874	0.606	1.261
Student Aid	0.826	0.509	1.342
Emergency Food	1.500	1.058	2.127

NOTE: The degrees of freedom in computing the confidence limits are 1713.
